# Prospective Study in Children with Complicated Urinary Tract Infection Treated with Autologous Bacterial Lysates

**DOI:** 10.3390/microorganisms9091811

**Published:** 2021-08-26

**Authors:** Ulises Hernández-Chiñas, María E. Chávez-Berrocal, Ricardo E. Ahumada-Cota, Armando Navarro-Ocaña, Luz M. Rocha-Ramírez, Yolanda Pérez-del Mazo, Maribel Alvarado-Cabello, Gabriel Pérez-Soto, Luis A. León-Alamilla, Salvador E. Acevedo-Monroy, Diego Esquiliano, Atlántida M. Raya-Rivera, Carlos A. Eslava

**Affiliations:** 1 Peripheral Unit of Basic and Clinical Research in Infectious Diseases, Public Health Department, Research Division, Faculty of Medicine Universidad Nacional Autónoma de México, Bacterial Pathogenicity Laboratory, Hemato-Oncology and Research Unit, Children’s Hospital of Mexico Federico Gómez, Dr. Márquez 162, Col. De los Doctores, Mexico City 06720, Mexico; ulisesh@unam.mx (U.H.-C.); malenachavezb@yahoo.com.mx (M.E.C.-B.); ricardoeahumada@gmail.com (R.E.A.-C.); salcevedom@hotmail.com (S.E.A.-M.); 2 Bacteriology Laboratory, Public Health Department, Faculty of Medicine, Universidad Nacional Autónoma de México, Avenida Universidad 3000, Ciudad Universitaria, Mexico City 04510, Mexico; arnava@unam.mx (A.N.-O.); gapeso@unam.mx (G.P.-S.); serologo@hotmail.com (L.A.L.-A.); 3 Unidad de Investigación en Enfermedades Infeccionas, Hospital Infantil de México Federico Gómez, Secretaría de Salud, Dr. Márquez 162, Col. Doctores, Mexico City 06720, Mexico; luzmrr7@yahoo.com.mx; 4 Bacterial Pathogenicity Laboratory, Hemato-Oncology and Research Unit, Children’s Hospital of Mexico Federico Gómez, Faculty of Medicine Universidad Nacional Autónoma de México, Dr. Márquez 162, Col. De los Doctores, Mexico City 06720, Mexico; yp498505@gmail.com (Y.P.-d.M.); cacho_wala17178@hotmail.com (M.A.-C.); 5 Tissue Engineering Laboratory, Children’s Hospital of Mexico Federico Gómez, Dr. Márquez 162, Col. De los Doctores, Mexico City 06720, Mexico; esquiliano@rocketmail.com

**Keywords:** UTI, *Escherichia coli*, UPEC, autologous bacterial lysates, autovaccine

## Abstract

Antimicrobial bacteria resistance is an important problem in children with recurrent urinary tract infections (rUTI), thus it is crucial to search for alternative therapies. Autologous bacterial lysates (ABL) may be a potential treatment for rUTI. Twenty-seven children with rUTI were evaluated for one year, urine and stool cultures were performed, 10 colonies of each culture were selected and those identified as *Escherichia coli* were characterized by serology. For patients who presented ≥10^5^ UFC/mL, an ABL was manufactured and administered orally (1 mL/day) for a month. Twelve children were monitored for ≥1-year, 218 urine and 11 stool samples were analyzed. *E. coli* (80.5%) was the main bacteria isolated from urine and feces (72%). *E. coli* of classical urinary serotypes (UPEC), O25:H4, O75:HNM, and O9:HNM were identified in patients with persistent urinary infection (pUTI). In 54% of patients treated with ABL, the absence of bacteria was observed in urine samples after 3 months of treatment, 42% of these remained without UTI between 10–12 months. It was observed that the use of ABL controlled the infection for almost 1 year in more than 60% of the children. We consider it necessary to develop a polyvalent immunogen for the treatment and control of rUTI.

## 1. Introduction

Urinary tract infections (UTI) are an important cause of disease during childhood, the condition may be acute or chronic and it is an indicator of anatomical and functional anomalies [1,2]. The vesicoureteral reflux (VUR), although of low degree in most cases, is identified in 25% of the first urinary infections and is related to rUTI and renal scars [2,3]. The infection is acquired by intestinal bacteria that ascend and colonize the peri-urethral, urethral epithelium and bladder (cystitis), sometimes reaching the ureter and kidney (pyelonephritis), or spreading through the bloodstream [4]. The opportune UTI diagnosis avoids complications such as urosepsis, urolithiasis, kidney abscess, and long-term issues of hypertension associated with kidney cortical scar and terminal kidney failure that may lead the patient to death unless a transplant is performed [2,3].

In most cases, UTI are caused by Gram-negative bacteria and less frequently by some Gram-positive bacteria. *Escherichia coli* (*E. coli*) is the most common pathogen involved in UTI cases in both children and adults; different studies infer that it is isolated in 80% of UTI community cases and in 60% of intra-hospital infections. [3,5,6]. *E. coli* strains have great genetic plasticity associated with the presence of different kinds of genes, which have contributed to the evolution of the bacteria through the generation of varieties of *E. coli* strains selected by environmental conditions. In this manner, there are strains defined as commensals that are part of the intestinal microbiota, other strains related to the etiology of intestinal diseases (DEC), and strains referred to as extraintestinal pathogens (ExPEC) [7,8]. The members of this last group are capable to colonize other habitats outside the gut and cause extraintestinal infections such as those of the urinary tract, therefore named uropathogenic *E. coli* (UPEC).

UPEC strains may reach the urethra, migrate from there and attach and invade the epithelium of the bladder and kidney; they also have mechanisms that allow them to evade the immunological system defenses and resist the effects of antimicrobials [4,9,10,11]. In addition to fimbriae, surface structures required for bacterial adherence, *E. coli* strains have flagella that contribute to the bacterium mobility, and also an important antigenic component. The use of anti O sera allowed the identification of serogroups O1, O2, O4, O6, O7, O15, O18, O25, O75, O83 and O175 which are most frequently related to UTI [12,13,14]. Additionally, the antigenic varieties of flagella (H) combined with the variety of O antigens define the serotypes of the *E. coli* group. So far O1:H4, O1:H6, O1:H7, O1:NM, O2:H1, O2:H4, O4:H5, O6:H1, O7:H4, O7:H6, O7:NM, O18ac:H7, O18ac:NM-, O25:H4, O75:NM comprised the most frequent serotypes associated with infections caused by UPEC strains [15,16].

The classic UTI treatment is antimicrobial therapy; however, the indiscriminate use of antimicrobial agents has led to the selection of multidrug-resistant UPEC strains that hinder the proper control of the infection, thus producing complicated UTI that are difficult to resolve. In some patients, it is possible that the strain that caused the primal infection is the same involved in the subsequent infections, for which these are called pUTI. In other patients, the responsible pathogen is different in each infectious process, thus defined as re-infections (riUTI) [17,18]. Regardless, in most cases, antimicrobial treatment is enough to solve most of the symptomatic UTI. The most frequently used antibiotics are trimethoprim-sulfamethoxazole (TMP-SMX), fluoroquinolones, nitrofurantoin, amoxicillin with or without clavulanic acid, second and third-generation cephalosporin and aminoglycosides [2,19,20,21]. In children with complicated UTI, the prophylactic use of antimicrobials such as nitrofurantoin or trimethoprim with sulfamethoxazole is common; however, there is evidence that this handling of the patient does not reduce renal scars and instead contributes to an increase in the selection of multidrug-resistant bacteria [2,19,20,21]. Given the possibility of severe complications such as kidney failure in children with a complicated UTI, it is important to develop useful treatments with no side effects.

The design of vaccines manufactured with UPEC strain immunogens has been proposed as an alternative for UTI antibiotic therapy [22,23,24,25]. However, a first infection of the urinary tract is not always enough to induce a protective immune response; additionally, the diversity of microorganisms, particularly *E. coli* strains associated with UTI, hinder the identification of an appropriate immunogen [26,27]. Immunization with a mix of complete inactivated or lysed microorganisms is a procedure used to induce a protective immune response. In this context some commercial vaccines have appeared (Uro-Vaxom^®^, Urovac^®^, ExPEC4V^®^), manufactured with lysates of different mixes of bacteria, including strains of *E. coli*, *Proteus vulgaris*, *Klebsiella pneumoniae*, *Morganella morganii* and *Enterococcus faecalis*, or bioconjugates created with different *E. coli* serotypes (O1A, O2, O6A y O25B) associated with different UTI cases [2,25,28]. Although these vaccines have been administered in different manners to treat UTI and clinical studies have been performed (Stage II and III), there have not been overwhelming results [29,30,31]. Autologous immunotherapy (autovaccine therapy) is another alternative that has been used for the treatment of infectious conditions incapable of resolution with antibiotic therapy. Autovaccines are considered a biological medicine that induces an active and protective immunization stimulating the production of G and M immunoglobulins and activation of T lymphocytes. Autovaccines are a lysate suspension manufactured with the isolated microorganism responsible for the infection and administered orally [32]. The aim of this work was to evaluate the use of ABL as an alternative treatment in children with complicated UTI through a prospective study; additionally, the pathogens associated with the infection and the characteristics of these were also evaluated.

## 2. Materials and Methods

### 2.1. Study Design

#### 2.1.1. Study Population

A prospective study over a 3-year period (2015–2018) was performed with 27 children diagnosed with recurrent (≥3 per year) UTI and with a prior surgical treatment due to anatomic anomaly and deficient response to antimicrobial treatment. All the children were outpatients of the Federico Gómez Children’s Hospital of Mexico, an institutional member of the National Health System of Mexico founded in 1943 and specializing in the treatment and control of pediatric ailments. Children with immune response alterations were excluded from the study. Sampling was consecutive and non-probabilistic where the number of patients analyzed relied on the cultures presenting bacteriuria (≥10^5^ UFC/mL) and on the agreement to receive bacterial lysate (BL) treatment. The research protocol was approved by the Research and Ethical Committee of Federico Gómez Children’s Hospital of Mexico (HIM/2014/022 SSA.1122). The parents of the children included in the study signed an informed consent letter with the previous approval statement by the child and none of the patients showed any side effects or adverse reactions following ABL administration.

#### 2.1.2. Biological Samples

Urine sampling was performed monthly according to traditional proceedings and delivered in less than 4 h to the Bacterial Pathogenicity Laboratory for analysis [33,34,35]. All urine samples were subjected to a urinalysis with a reactive test strip for 10 parameters (Mission, San Diego, CA, USA) and a bacterial culture. Additionally, a group of randomly selected children also had a stool sample requested.

#### 2.1.3. Bacterial Culture

Bacterial counts were performed spreading 100 µL of urine on Luria–Bertani agar (DIBICO, Mexico City, Mexico) and for bacterial isolation, urine sediment was streaked on Blood agar (DIBICO, Mexico City, Mexico) with 5% sheep blood and MacConkey agar (BD Bioxon, Cuatitlán Izcalli, Mexico). For fecal samples, a fraction of feces was taken and inoculated in MacConkey agar (BD Bioxon, Cuatitlán Izcalli, Mexico). All plates were incubated at 37 °C for 24 h, and afterward, 10 colonies were randomly selected and recovered from all positive urine samples (≥10^5^ CFU/mL) and fecal cultures. Finally, each colony was subjected to a Gram stain test and the IMViC biochemical battery for the identification of *Enterobacteriaceae* [36].

#### 2.1.4. Serotyping of *E. coli* Isolates

Strains identified as *E. coli* were serotyped by agglutination assays using 96-well microtiter plates and rabbit antisera against O1 to O187 somatic (O) antigens and 53 flagellar (H) antigens prepared in rabbits (SERUNAM, registered trademark in Mexico, with number 323,158/2015) using the method described by Orskov and Orskov [37] with minor modifications. The patient was colonized by the isolated strain when ≥8 out of 10 colonies belonged to the same serotype.

#### 2.1.5. Autologous Bacterial Lysate (ABL)

The ABL was manufactured using the isolated strain from the patient urine culture, using the protocol described by Ahumada-Cota et al., 2020 [33]. In brief, strains were streaked by massive growth on Luria–Bertani agar in 10 assay tubes (16 × 150) and incubated at 37 °C for 18 h; from these cultures, using saline solution (0.85%) a bacterial suspension of 80 mL with a final concentration of 10^8^ CFU/mL was prepared. The suspension was inactivated by effluent steam (110 °C for 1 h), centrifuged (4600× *g*) and sterilized by filtration (0.22 µm; Merc-Millipore, Tullagreen, Carrigtwohill, Co.Cork, Ireland). The filtered solution was dosed in 8 vials (10 mL/vial) and a sterility test by incubation (37 °C for 24 h) was performed. An oral dose of 1 mL per day was indicated to the patient and administered with the help of a sterile syringe under aseptic conditions. Afterward, if the urine culture was negative, the same ABL was administered to the patient for two additional months, and if the urine culture was positive, a new ABL was prepared and administered to the patient, with the same specifications.

## 3. Results

### 3.1. Clinical Characteristics of the Patients

From April 2014 to November 2019, 25 girls and 2 boys between the ages of 5 and 17 years diagnosed with complicated UTI, were recruited for this study. Patients were monitored over a period of 2 to 39 months (an average of 16.8 ± 11.8 SD); at the beginning of the trial, 25 children reported cystitis, one reported pyelonephritis and another one asymptomatic bacteriuria. The most frequent clinical diagnoses were bladder dysfunction (30%), Hinman Syndrome (i.e., urinary voiding dysfunction in the neurologically intact child), and neurogenic bladder (13%). Only 16 (59%) children could be monitored for ≥1 year, 12 of these patients provided a urine sample more than 6 times in a 1-year period (Table S1).

### 3.2. Urinalysis

The results of urinalysis of patients with riUTI and pUTI showed significant differences in Protein, Leukocyte esterase and Nitrites. Proteins were detected in a range from 0–15 mg/dL to 0–30 mg/dL with a mode of 70 mg/dL and 0 mg/dL in patients with pITU and rITU, respectively. Leukocyte esterase showed a detection interval of 0–500 Leu/µL in both groups with a mode of 70 Leu/µL in pUTI and no detection in rUTI. Nitrite detection was 63% and 48.6% in patients with pUTI and rUTI, respectively, with a value of *p* > 0.05.

### 3.3. UTI Etiology

A total of 218 urine cultures were analyzed, where 131 (61%) had a bacterial count of ≥10^5^ CFU/mL. From the 1310 colonies recovered, the most frequent isolates were *E. coli* (80.5%), while other bacteria identified were *Proteus* spp. (3.4%), *Citrobacter* spp. (3%), *Enterobacter* spp. (3%), *Pseudomonas aeruginosa* (1.5%), and *Klebsiella* spp. (0.7%). Additionally, in 10 (7.6%) urine cultures Gram-positive bacteria were isolated. Notably, only one bacterial species was identified in 99% of the urine cultures.

### 3.4. E. coli Serotyping

A total of 38 different serogroups in 820 (78%) *E. coli* isolates were identified, nine classic and 29 non-classic UPEC serogroups were identified in 440 (41.7%) and 380 (36%) strains, respectively; statistical analysis showed no significant difference between both groups (*p* > 0.05). The data show that the most frequently isolated serogroups were O25, O75 and O9. In the 235 (22.3%) *E. coli* isolates in which the somatic antigen was not defined, 70 (30%) presented rough colony morphology and 165 (70%) did not agglutinate with any sera and were defined as non-typeable (Table 1). Serotype was identified for 930 (88%) *E. coli* isolates, while the remaining 125 (12%) could not be typified. A total of 56 different serotypes were identified, 7 considered classic UPEC (O1:NM, O1:H7, O4:H5, O6:H1, O7:H4, O25:H4, O75:HNM) identified in 280 (30%) strains and 49 non-classic UPEC in 650 (70%) strains. Analysis of non-classic serotypes showed 7 serotypes associated with enteropathogenic *E. coli* (EPEC) (O101:NM, O109:H21, O23:NM, O45:NM, O7:NM), enterotoxigenic (ETEC) (O25:NM) and enteroaggregative (EAEC) (O73:H18) pathotypes isolated from 7 urine cultures belonging to different patients (Figure S1).

### 3.5. Reinfections and Persistence

*E. coli* was isolated in 80% of the total samples; conversely, we consider that antigenic characterization would allow us to identify whether an infection was caused by a previous strain or by a different one. The presence of the same serotype (O25:H4, O9:HNM and O75:HNM) was observed in the urine cultures of three (11.2%) patients over more than six months, which means that these were pUTI. In the remaining 24 children (88.8%) the serological analysis showed different *E. coli* serotypes in each analyzed sample, which confirms that the process is reinfection (Figure S1). Although classic UPEC serogroups were identified in both groups of patients (reinfection vs. persistence), the statistical analysis only showed meaningful differences (*p* < 0.05) for the O75 and O9 serogroups.

### 3.6. E. coli Strains Recovered from Feces

Stool samples from 7 patients were analyzed and a total of 11 cultures were performed, where *E. coli* was identified in 73% (80/110) of the isolated colonies, an average of 7 isolates per sample (Table 2). A total of 34 different serotypes were identified, an average of three *E. coli* antigenic varieties per stool culture; however, the stool of two patients (NBA and RMR) showed the presence of a dominant serotype (O6:H1 and O25:H4). Additionally, the serotype analysis (Table 2) revealed 51% (41/80) of the *E. coli* strains belonged to the classic UPEC serotypes O6:H1, O7:H4, O1:H6, O4:H5, O25:H4, O51:HNT, O34:HNM, O8:H7; while 5% (4/80) of the isolates included DEC pathotype strains: EPEC (O45:HNM, O76:HNM), EAEC (O20:H30) and EIEC (O164:HNM). The remaining 35 strains belonged to none of the *E. coli* pathogenic groups, suggesting these strains are associated with the commensal microbiota. A comparison between the *E. coli* serotypes isolated from urine and stool samples from the same patients revealed that only three (ARV, VR and RMR) individuals presented the same serotype in both samples.

### 3.7. Effect of the Autologous Bacterial Lysates (ABL) in Children with Complicated UTI

**Figure 1:**
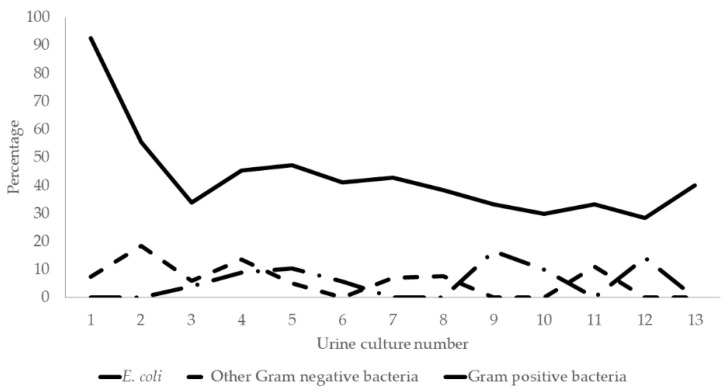
*E. coli* and other bacteria isolated from urine samples from children with complicated UTI and treated with ABL.

All children in this study presented a complicated UTI with poor response to antimicrobial treatment; as an alternative therapy, these children received an ABL administered orally for a month. To evaluate the immunostimulatory response of the ABL the results obtained from the second urine culture were considered. At the beginning of the study, *E. coli* was the uropathogen present in 25 (92.5%) patients and the effect of the ABL was addressed by the number of positive *E. coli* cultures that were obtained in subsequent urine samples. The number of positive cultures went down to 55.5% and 34% for the second and third months, respectively. Urine cultures remained negative on a 38% average from the 4th to the 13th sample (Figure 1). Remarkably, seven patients changed the etiology of the UTI from *E. coli* in an event (i.e., a specific sample) to a different strain (*Citrobacter* spp., *Proteus* spp. or Gram-positive coccus) in the next one (Figure S1).

**Figure 2:**
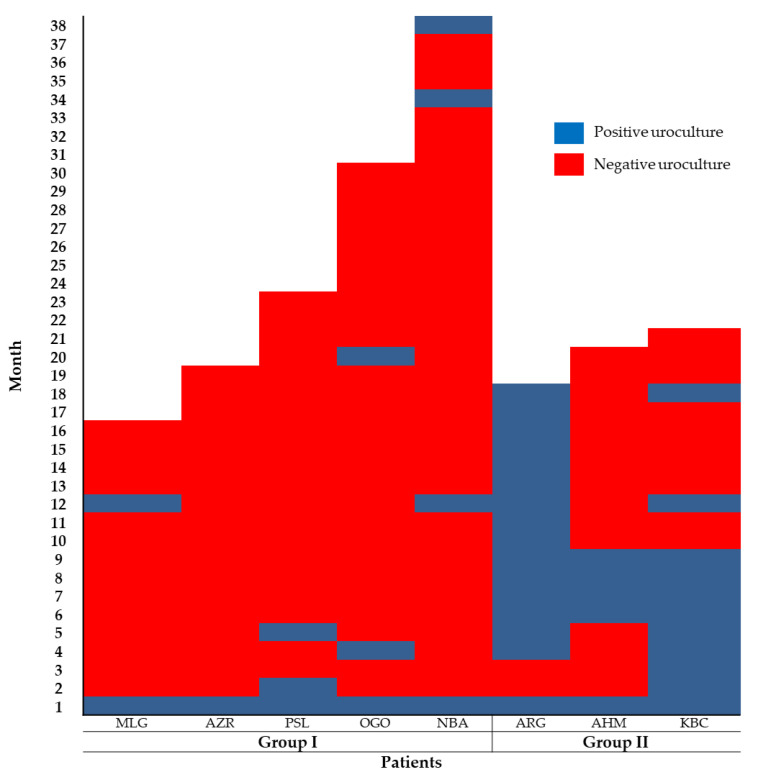
Protection induced by the ABL analyzed monthly by urine culture. Group I, five patients: MLG, 16 months stay and one reinfection at month 12; AZR, 19 months stay and negative urinary cultures from the two first months of treatment; PSL, 23 months stay and one reinfection at month 5; OGO, 30 months stay and reinfections at months 4 and 20; NBA, 38 months stay and reinfections at months 2, 34 and 38. Group II, three patients: ARG, 18 months stay and no bacteriuria at months 2 and 3; AHM, 20 months stay and reinfections at months 6 and 9; KBC, 21 months stay and sustained bacteriuria during the first 9 months in the study and at months 12 and 18.

The efficacy of the ABL was assessed with the data from 12 (44%) patients that could be monitored for ≥1 year and with ≥6 urine cultures analyzed. For this analysis, the patients were divided into three categories: the first group (I) consisted of five patients who received ABL for 3 months and showed a continuous improvement, ranging from 7 to 11 consecutive negative urine cultures; the second group (II) included three patients who showed between 2 and 4 negative urine cultures (Figure 2); additionally, there were four patients (group III) who always had positive urine cultures. Notably, one of the patients in group I presented bacteriuria two months after the beginning of the trial, while the rest remained asymptomatic and with no bacteriuria until the 12th month. However, in the 13th month, bacteriuria was identified in two patients where urine culture showed the presence of *E. coli*; after which a new ABL was manufactured and administered for the next 2 months, these two patients sustained negative urine cultures for 4 and 10 more months, respectively. The remaining children showed no evidence of bacteriuria for the rest of their time in the trial, i.e., 18th and 23rd month, respectively. Patients in group II presented bacteriuria in the third month and sustained the UTI until the 12th month. After a year of treatment, two patients in this group did not show infections for a lapse of 5 and 8 months, respectively. (Figure 2).

## 4. Discussion

To avoid complications that may damage the physical and emotional integrity of the patient, diseases, in general, need an opportune diagnosis and effective treatment. UTI appear at all stages of life and affect very young children as well as older adults. In 2018, UTI were the third most common cause of disease in Mexico with 4,339,674 cases reported, where 3,343,709 (77%) occurred in women. Additionally, age-based analysis of the same report showed that 320,039 (10%) cases happened in girls from under one year old and up to 14 years old, which makes this infection one of the most frequent diseases during the pediatric stage. The high economic costs and clinical collateral effects of UTI are constant worldwide [3]. A patient is considered to have a rUTI when there are ≥2 episodes of UTI in the upper tracts (pyelonephritis), a high UTI episode and a low UTI episode (cystitis), or ≥3 episodes of cystitis within a year. Different risk factors have been associated with rUTI in children, these include anatomical abnormalities, neurological abnormalities, or intestinal dysfunction, all of which increase the risk for kidney scarring [3,38]. Obstructive uropathies and VUR hinder the normal urinary flux, relevant for the elimination of microorganisms present in the urinary tract, thereby significantly increasing the development of rUTI [1]. The present work reports data from a follow-up study conducted in 27 children with complicated UTI, associated with urinary dysfunction, Hinman syndrome, and neurogenic bladder, disorders in which VUR is a constant. As mentioned, these clinical syndromes are the main cause of rUTI and are associated with permanent kidney damage and complications such as hypertension and chronic renal insufficiency and in worst cases scenarios leading to a kidney transplant [1,3].

Herein, a total of 218 samples were analyzed, where 131 presented bacteriuria (≥10^5^ CFU/mL) and the urine analysis with leukocyte esterase and presence of nitrites confirmed the UTI diagnosis associated with the presence of Gram-negative bacteria. Ten colonies were selected from each culture, if ≥8 out of 10 isolates were identified as the same bacteria, the patient was considered colonized. This criterion was used because the samples may have been contaminated when collected, and the presence of different microorganisms was possible [34]. It is well known that rUTI symptoms are associated with different types of enterobacteria, and in this study, the results confirmed the previously reported and showed, using metabolic tests and serotyping, that *E. coli* was the most common uropathogen [1,36,37]. The result was expected because *E. coli* is a member of the intestinal biota, and it has been reported that this situation is an independent risk factor for bacteriuria of which its main source is the contamination of the periurethral area with intestinal biota [39,40,41]. An important aspect of this research was the phenotype characterization through the serotype analysis of the *E. coli* strains isolated from urine and stool samples. Using specific sera against O and H antigens, it was confirmed that children were colonized by a specific strain since different isolates from the same sample presented the same serotype. We were able to identify and establish the great diversity of serogroups and serotypes in 78% of *E. coli* strains associated with UTI. When serogroups were evaluated, 42% of isolates matched those serogroups defined as classic UPEC, where O25 and O75 were the most frequents; however, the rest belonged to other serogroups, some of which were included in the DEC pathotype, although statistical analysis of both groups showed no significant differences. Some of the non-typeable strains showed rough morphology and others did not agglutinate with any of the 186 sera used in the test. In two community UTI studies conducted in Mexico, only UPEC serogroups were identified using PCR, where O25 and O75 serogroups were the most frequent. It is important to point out that in both studies only classic UPEC serogroups could be detected because PCR primers were designed only for the identification of this group; under these circumstances, it is not possible to reach a conclusion [42,43]. On the other hand, Sharma et al. [44] using polyvalent sera were able to typify 44% *E. coli* strains isolated from patients with UTI, similar to the results reported here. In the same study, the classic UPEC serogroups O15 and O75 were identified as the most frequent; however, the rest of those isolates could not be identified. Altogether, the results reported here and in the mentioned studies showed that riUTI can be caused by different serogroups and not only by the so-called classic UPEC; therefore, it can be assumed that there are antigenic varieties of UPEC that are specific to each region and may be considered as native [45]. It is known that environmental factors and diet impact the composition of the intestinal microbiota, an important element when considering the great diversity of *E. coli* strains related to the etiological pathogenesis of UTI worldwide [46,47].

UTI are classified as recurrent when symptoms of infection appear with a frequency of ≥3 in one year or ≥2 within a period of six months. rUTI may appear as relapsed when the same microorganism is isolated after receiving specific treatment or as reinfections if the causing agent is different from the one responsible for the previous UTI symptoms [17]. Conversely, there is a greater risk of rUTI development in children with vesical-urinary reflux; in this study, the main clinical disorders observed in the children (bladder dysfunction, Hinman Syndrome, and neurogenic Bladder) were associated with this clinical manifestation [1,3,38]. As mentioned before, three children presented persistent UTI caused by classical UPEC serotypes while the remaining 24 showed a variety of *E. coli* serotypes in 24 children. This is relevant because the classical UPEC group presents specific genotypical features like genes favoring adherence and invasiveness to the bladder cells, resistance to sera, and biofilm production, abilities that UPEC strains use to stay over long periods [14]. To corroborate the participation of periurethral contamination as the UTI source, the analysis of a stool sample from some seven children was included where three patients showed the same *E. coli* serotype in both urine and stool samples. These strong results prove that urethra contamination by fecal *E. coli* is the source of UTI, which along with the anatomical alterations of the urinary tract contribute to the presence of recurrent and persistent UTI [39,40,41]. Another interesting fact is the presence of strains belonging to DEC serotypes in urine samples, preliminary results in these strains revealed the presence of UPEC associated genes, the existence of these hybrids is probably associated with the horizontal transfer of genes [48]. Due to the ineffective and even counterproductive prophylactic use of antimicrobials for the treatment and control of rUTI, as is the case for the patients herein analyzed, the need for a safe, effective, and acceptable alternative therapy has been proposed. The use of probiotics and other compounds such as cranberry juice has been proposed for this purpose and commercial immunostimulants such as Urovaxom^®^ have been designed to eliminate bacteriuria in adult patients with rUTI [2,25,49,50,51,52]. However, these types of immunogens are manufactured with bacteria that are different from the ones causing UTI in other parts of the world, a key aspect as we show in this study. For this reason, we believe it is important to employ region-specific bacteria for the manufacturing of the immunogens. The use of orally administered ABL for the treatment and prevention of UTI has been documented since the 1940s and its use is recommended for rUTI in the treatment guidelines of the European Union [2,25,32]. In a study conducted in our laboratory [33], the use of ABL for the treatment and control of rUTI in adults was evaluated and efficacy of approximately 70% in controlling the recurrence of the symptoms was observed. In the present study, an efficacy of 50% was observed, a lower result than what was reported for adults; these differences might be due to the functional alterations that the children have in their urinary tracts or to specific features of the strains causing the symptoms; it is known that UPEC strains can invade the epithelium of the bladder and to remain in a quiescent state, serving as a reserve for recurrent infections [11]. In 7 of the 12 patients that could be monitored for a 1-year period the presence of bacteria was negative after the second month and they remained asymptomatic with negative cultures for a minimum period of 5 months. Patients with a new episode of bacteriuria were given a new ABL, prepared with the new isolate, it was observed that with this reinforcement the infection was controlled after the second month of administering treatment. The commercial lysate Urovaxom^®^ treatment stipulates a new immunization between 6 and 9 months [25], which correlated with the observations made in this study, the latter could be related to the alterations presented by the patients in this study. Because each patient was under their own control, children were administered antimicrobials as well as ABL; however, the patients showed rUTI for months prior to the ABL, this allowed us to assure the positive effect of administering the ABL. Immunopharmacological studies with humans have shown that bacterial lysates increase the synthesis of serum interferon and of urine secreting IgA, as well as the number of active T lymphocytes [29,53]. The stimulus favors the activation of immune defense in animal and human models, the stimulation of the lymphoid tissue associated with the intestine could induce a general response over the whole mucosa [associated lymphoid tissue (MALT), which is found in the digestive, respiratory and genitourinary tracts [54].

In some patients, bacteriuria could not be resolved with the ABL treatment, the strains associated with these persistent UTI belonged to the serotypes O25:H4, O75:NM and O9:HNM, all defined as classic UPEC [13,33,38,42,55]. Studies where different properties of these serotypes were analyzed infer that *E. coli* O25:H4 strains exhibit different virulent types, showing resistance to a variety of antimicrobials (MDR) and having the ability to adhere and invade white blood cells, features that favor these strains to enter in a quiescent state (latent), which contributes to the continued infection of the bladder [13,56]. Moreover, O75:NM (Non-Mobile) strains have been reported as serum resistant, a feature linked to the evasion of the complement system [57]; also, this is the first work where the O9:NM serotype was associated with a persistent UTI. All these remarkable results stress the importance of genotypical characterization of the isolated strains to know which genes might play a part in the virulence of the UPEC in persistent UTI.

## 5. Conclusions

In conclusion, serotyping of *E. coli* isolates from UTI proved to be a useful tool since the characterization of the strains allowed us to observe that there is more than one antigenic variety of UPEC present in some UTI; this could be a factor in treatment failures. Additionally, serotyping also allowed the identification of the most common UPEC strains in Mexican children; this information, along with the previously UPEC identification described in adults, indicates which strains could be used in the manufacturing of a polyvalent vaccine. The administration of ABL was effective for the treatment and control of UTI in children with complicated infections, and this has great relevance since ABL could significantly contribute to patients who do not experience substantially improved clinical conditions after surgical treatment and antimicrobials.

## Figures and Tables

**Table 1:** *E. coli* serogroups identified in urine from patients with recurrent and persistent UTI.

UPEC Classification	Serogroup ^‡^	No. of Patients *	No. of Isolates *	*n* (%)
Classic	O25	6	160	440 (41.7)
	O1 and O75 *	3	130	
	O8 and O9 *	2	100	
	O2, O4, O6 and O7	1	50	
Non-classic	O11, O17 and O102	3	75	380 (36)
	O57, O96 and O170	2	65	
	O12. O20, O23, O29, O32, O35, O45, O49, O73, O100, O101, O105, O109, O124, O144, O147, O153, O154, O164, O174, O178, O49766 and O684474	1	240	
Non-Typeable	OND ^‡^	7	165	235 (22.3)
	OR ^‡^	6	70	

* Serogroup associated to persistent UTI: O25 to patient RMR, O75 to patient AA and O9 to patient KMB. ^‡^ NM, non-motile; ND, non-determined; OR, rough phenotype.

**Table 2:** Serotypes and pathogenic groups identified in *E. coli* strains isolated from urine and stool samples from children with rUTI.

Patients	Urine Cultures	Serotypes (No. of Isolated Bacteria)	Stool Cultures	Serotype (No. of Isolated Bacteria)
NBA	5	O45:NM (10), O35:H10 (10), O64474:HNM (10), O96:H16 (8), OR:HNM (2) and O105:H18 (10)	1	O6:H1 (8) *
ARV	2	O11:H25 (10), O23:HNM (6) and **O7:H4 (4)**	1	**O7:H4 (2)** *, OND:NM (3) *, O1:H6 (2) *
VR	2	**O4:H5 (10)** and OR:H15 (10)	2	O169:H ND (4), **O4:H5 (2)** *, O45:HNM (1) ^‡^, O157:HND (3), OND:NM (3) and O138:H34 (1)
AMR	2	O25:H4 (12) and O17:H18(8)	1	O48:HND (8)
RMR	7	**O25:H4 (70)**	3	OND:H16 (2), OND:HND (1), O89:H38(1), **O25:H4 (8)** *, O25:H10 (1), O29:H10 (1), O51:HND (3) *, OR:HNM (2) and O129:H30 (1)
AA	12	O75:HNM (90)	1	O28ab:H7 (2), O34:NM (1) *, O8:H7 (5) *, O20:H30 (1) ^‡^
OGO	14	O170:HNM (10) and O25:H4 (10)	2	O3:H14 (1), O18ac:H14 (2), O21:H14 (1), O128ab:H5 (2), O154:H4 (1), O164:HNM (1) ^‡‡^, O128ac:HNM (1), OND:HNM (3), OR:HNM (1), O76:HNM (1) **

The serotypes identified in urine and feces in the same patient are indicated in bold. ND: Serotype not included in the typing scheme. NM: Non motile, HND: Flagellar antigen (H) Non-Determined with the 56-serum tested. * UPEC serotype, ** EPEC serotype, ^‡^ EAEC serotype, ^‡‡^ EIEC serotype.

## Data Availability

The data presented in this study are available on request from the corresponding author.

## Supplementary Materials

The following are available online at https://www.mdpi.com/article/10.3390/microorganisms9091811/s1, Figure S1: Bacterial strains isolated from urine samples and *E. coli* serotypes identified during the prospective study of children with UTI. Table S1: Patients’ months of stay and number of positive cultures.

## References

[1] Tullus K., Shaikh N. (2020). Urinary tract infections in children. Lancet.

[2] Khan A., Jhaveri R., Seed P.C., Arshad M. (2019). Update on Associated Risk Factors, Diagnosis, and Management of Recurrent Urinary Tract Infections in Children. J. Pediatr. Infect. Dis. Soc..

[3] Millner R., Becknell B. (2019). Urinary Tract Infections. Pediatr. Clin..

[4] Asadi Karam M.R., Habibi M., Bouzari S. (2019). Urinary tract infection: Pathogenicity, antibiotic resistance and development of effective vaccines against Uropathogenic *Escherichia coli*. Mol. Immunol..

[5] Flores-Mireles A.L., Walker J.N., Caparon M., Hultgren S.J. (2015). Urinary tract infections: Epidemiology, mechanisms of infection and treatment options. Nat. Rev. Microbiol..

[6] Oliveira E.A., Mak R.H. (2020). Urinary tract infection in pediatrics: An overview. J. Pediatr..

[7] Russo T.A., Johnson J.R. (2000). Proposal for a New Inclusive Designation for Extraintestinal Pathogenic Isolates of *Escherichia coli*: ExPEC. J. Infect. Dis..

[8] Kaper J.B., Nataro J.P., Mobley H.L.T. (2004). Pathogenic *Escherichia coli*. Nat. Rev. Microbiol..

[9] Ejrnæs K. (2011). Bacterial characteristics of importance for recurrent urinary tract infections caused by *Escherichia coli*. Dan. Med. Bull..

[10] Blango M.G., Mulvey M.A. (2010). Persistence of Uropathogenic *Escherichia coli* in the Face of Multiple Antibiotics. Antimicrob. Agents Chemother..

[11] Mulvey M.A., Schilling J.D., Martinez J.J., Hultgren S.J. (2000). Bad bugs and beleaguered bladders: Interplay between uropathogenic *Escherichia coli* and innate host defenses. Proc. Natl. Acad. Sci. USA.

[12] Noie Oskouie A., Hasani A., Ahangarzadeh Rezaee M., Soroush Bar Haghi M.H., Hasani A., Soltani E. (2019). A Relationship Between O-Serotype, Antibiotic Susceptibility and Biofilm Formation in Uropathogenic *Escherichia coli*. Microb. Drug Resist..

[13] Shokouhi Mostafavi S.K., Najar-Peerayeh S., Mohabbati Mobarez A., Kardoust Parizi M. (2019). Serogroup distribution, diversity of exotoxin gene profiles, and phylogenetic grouping of CTX-M-1- producing uropathogenic *Escherichia coli*. Comp. Immunol. Microbiol. Infect. Dis..

[14] Gao Q., Zhang D., Ye Z., Zhu X., Yang W., Dong L., Gao S., Liu X. (2017). Virulence traits and pathogenicity of uropathogenic *Escherichia coli* isolates with common and uncommon O serotypes. Microb. Pathog..

[15] Terai A., Yamamoto S., Mitsumori K., Okada Y., Kurazono H., Takeda Y., Yoshida O. (1997). *Escherichia coli* Virulence Factors and Serotypes in Acute Bacterial Prostatitis. Int. J. Urol..

[16] Bidet P., Mahjoub-Messai F., Blanco J., Blanco J., Dehem M., Aujard Y., Bingen E., Bonacorsi S. (2007). Combined Multilocus Sequence Typing and O Serogrouping Distinguishes *Escherichia coli* Subtypes Associated with Infant Urosepsis and/or Meningitis. J. Infect. Dis..

[17] Dason S., Dason J.T., Kapoor A. (2011). Guidelines for the diagnosis and management of recurrent urinary tract infection in women. Can. Urol. Assoc. J..

[18] Malik R.D., Wu Y.R., Zimmern P.E. (2018). Definition of Recurrent Urinary Tract Infections in Women: Which One to Adopt?. Female Pelvic Med. Reconstr. Surg..

[19] Bader M.S., Loeb M., Brooks A.A. (2017). An update on the management of urinary tract infections in the era of antimicrobial resistance. Postgrad. Med..

[20] Behzadi P., Urbán E., Matuz M., Benkő R., Gajdács M. (2020). The Role of Gram-Negative Bacteria in Urinary Tract Infections: Current Concepts and Therapeutic Options. SpringerLink.

[21] Buettcher M., Trueck J., Niederer-Loher A., Heininger U., Agyeman P., Asner S., Berger C., Bielicki J., Kahlert C., Kottanattu L. (2020). Swiss consensus recommendations on urinary tract infections in children. Eur. J. Pediatr..

[22] Schmidhammer S., Ramoner R., Höltl L., Bartsch G., Thurnher M., Zelle-Rieser C. (2002). An *Escherichia coli*-based oral vaccine against urinary tract infections potently activates human dendritic cells. Urology.

[23] Hopkins W.J., Elkahwaji J., Beierle L.M., Leverson G.E., Uehling D.T. (2007). Vaginal Mucosal Vaccine for Recurrent Urinary Tract Infections in Women: Results of a Phase 2 Clinical Trial. J. Urol..

[24] Brumbaugh A.R., Mobley H.L. (2012). Preventing urinary tract infection: Progress toward an effective *Escherichia coli* vaccine. Expert Rev. Vaccines.

[25] Prattley S., Geraghty R., Moore M., Somani B.K. (2020). Role of Vaccines for Recurrent Urinary Tract Infections: A Systematic Review. Eur. Urol. Focus.

[26] Billips B.K., Yaggie R.E., Cashy J.P., Schaeffer A.J., Klumpp D.J. (2009). A Live-Attenuated Vaccine for the Treatment of Urinary Tract Infection by Uropathogenic *Escherichia coli*. J. Infect. Dis..

[27] Sivick K.E., Mobley H.L.T. (2010). Waging War against Uropathogenic *Escherichia coli*: Winning Back the Urinary Tract. Infect. Immun..

[28] Aziminia N., Hadjipavlou M., Philippou Y., Pandian S.S., Malde S., Hammadeh M.Y. (2019). Vaccines for the prevention of recurrent urinary tract infections: A systematic review. BJU Int..

[29] Huber M., Baier W., Serr A., Bessler W. (2000). Immunogenicity of an *E. coli* extract after oral or intraperitoneal administration: Induction of antibodies against pathogenic bacterial strains. Int. J. Immunopharmacol..

[30] Bauer H.W., Rahlfs V.W., Lauener P.A., Blessmann G.S.S. (2002). Prevention of recurrent urinary tract infections with immuno-active *E. coli* fractions: A meta-analysis of five placebo-controlled double-blind studies. Int. J. Antimicrob. Agents.

[31] Taha Neto K.A., Nogueira Castilho L., Reis L.O. (2016). Vacuna oral (OM-89) en la profilaxis de infección urinaria recurrente: Una revisión sistemática realista con metaanálisis. Actas Urol. Esp..

[32] Cazzola M., Anapurapu S., Page C.P. (2011). Polyvalent mechanical bacterial lysate for the prevention of recurrent respiratory infections: A meta-analysis. Pulm. Pharmacol. Ther..

[33] Ahumada-Cota R.E., Hernandez-Chiñas U., Milián-Suazo F., Chávez-Berrocal M.E., Navarro-Ocaña A., Martínez-Gómez D., Patiño-López G., Salazar-Jiménez E.P., Eslava C.A. (2020). Effect and Analysis of Bacterial Lysates for the Treatment of Recurrent Urinary Tract Infections in Adults. Pathogens.

[34] LaRocco M.T., Franek J., Leibach E.K., Weissfeld A.S., Kraft C.S., Sautter R.L., Baselski V., Rodahl D., Peterson E.J., Cornish N.E. (2016). Effectiveness of Preanalytic Practices on Contamination and Diagnostic Accuracy of Urine Cultures: A Laboratory Medicine Best Practices Systematic Review and Meta-analysis. Clin. Microbiol. Rev..

[35] Wilson M.L., Gaido L. (2004). Laboratory Diagnosis of Urinary Tract Infections in Adult Patients. Clin. Infect. Dis..

[36] Barrow G.I., Feltham R.K.A. (1993). Characters of Gram-negative bacteria. Cowan and Steel’s Manual for the Identification of Medical Bacteria.

[37] Orskov F., Orskov I. (1992). *Escherichia coli* serotyping and disease in man and animals. Can. J. Microbiol..

[38] Tewary K., Narchi H. (2015). Recurrent urinary tract infections in children: Preventive interventions other than prophylactic antibiotics. World J. Methodol..

[39] Vosti K.L. (2007). A prospective, longitudinal study of the behavior of serologically classified isolates of *Escherichia coli* in women with recurrent urinary tract infections. J. Infect..

[40] Magruder M., Sholi A.N., Gong C., Zhang L., Edusei E., Huang J., Albakry S., Satlin M.J., Westblade L.F., Crawford C. (2019). Gut uropathogen abundance is a risk factor for development of bacteriuria and urinary tract infection. Nat. Commun..

[41] Matsui Y., Hu Y., Rubin J., de Assis R.S., Suh J., Riley L.W. (2020). Multilocus sequence typing of *Escherichia coli* isolates from urinary tract infection patients and from fecal samples of healthy subjects in a college community. MicrobiologyOpen.

[42] Paniagua-Contreras G.L., Monroy-Pérez E., Rodríguez Moctezuma J.R. (2017). Virulence factors, antibiotic resistance phenotypes and O-serogroups of *Escherichia coli* strains isolated from community-acquired urinary tract infection patients in Mexico. J. Microbiol. Immunol. Infect..

[43] Hernández-Chiñas U., Pérez-Ramos A., Belmont-Monroy L., Chávez-Berrocal M.E., González-Villalobos E., Navarro-Ocaña A., Eslava C.A., Molina-Lopez J. (2019). Characterization of auto-agglutinating and non-typeable uropathogenic *Escherichia coli* strains. J. Infect. Dev. Ctries..

[44] Sharma S., Kaur N., Malhotra S., Madan P., Ahmad W., Hans C. (2016). Serotyping and Antimicrobial Susceptibility Pattern of *Escherichia coli* Isolates from Urinary Tract Infections in Pediatric Population in a Tertiary Care Hospital. J. Pathog..

[45] Aragón I.M., Herrera-Imbroda B., Queipo-Ortuño M.I., Castillo E., Moral J.S.-G.D., Gómez-Millán J., Yucel G., Lara M.F. (2018). The Urinary Tract Microbiome in Health and Disease. Eur. Urol. Focus.

[46] Duriez P., Clermont O., Bonacorsi S., Bingen E., Chaventré A., Elion J., Picard B., Denamur E. (2001). Commensal *Escherichia coli* isolates are phylogenetically distributed among geographically distinct human populations. Microbiology.

[47] Yu M., Li Z., Chen W., Rong T., Wang G., Ma X. (2019). Microbiome-Metabolomics Analysis Investigating the Impacts of Dietary Starch Types on the Composition and Metabolism of Colonic Microbiota in Finishing Pigs. Front. Microbiol..

[48] Lindstedt B.-A., Finton M.D., Porcellato D., Brandal L.T. (2018). High frequency of hybrid *Escherichia coli* strains with combined Intestinal Pathogenic *Escherichia coli* (IPEC) and Extraintestinal Pathogenic *Escherichia coli* (ExPEC) virulence factors isolated from human faecal samples. BMC Infect. Dis..

[49] Schwenger E.M., Tejani A.M., Loewen P.S. (2015). Probiotics for preventing urinary tract infections in adults and children. Cochrane Database Syst. Rev..

[50] O’Brien V.P., Hannan T.J., Nielsen H.V., Hultgren S.J. (2016). Drug and Vaccine Development for the Treatment and Prevention of Urinary Tract Infections. Microbiol. Spectr..

[51] Leung A.K.C., Wong A.H.C., Leung A.A.M., Hon K.L. (2019). Urinary Tract Infection in Children. Recent Pat. Inflamm. Allergy Drug Discov..

[52] Brodie A., El-Taji O., Jour I., Foley C., Hanbury D. (2020). A Retrospective Study of Immunotherapy Treatment with Uro-Vaxom (OM-89^®^) for Prophylaxis of Recurrent Urinary Tract Infections. Curr. Urol..

[53] Rosenthal V. (1986). Effect of a Bacterial Extract on Cellular and Humoral Immiine Responses in Humans. Immunopharmacol. Immunotoxicol..

[54] Naber K.G., Cho Y.-H., Matsumoto T., Schaeffer A.J. (2009). Immunoactive prophylaxis of recurrent urinary tract infections: A meta-analysis. Int. J. Antimicrob. Agents.

[55] Rogers B.A., Sidjabat H.E., Paterson D.L. (2011). *Escherichia coli* O25b-ST131: A pandemic, multiresistant, community-associated strain. J. Antimicrob. Chemother..

[56] Barrios-Villa E., Cortés-Cortés G., Lozano-Zaraín P., de la Paz Arenas M.M., de la Peña C.F., Martínez-Laguna Y., Torres C., del Carmen Rocha-Gracia R. (2018). Adherent/invasive *Escherichia coli* (AIEC) isolates from asymptomatic people: New *E. coli* ST131 O25:H4/H30-Rx virotypes. Ann. Clin. Microbiol. Antimicrob..

[57] Nimmich W., Voigt W., Seltmann G. (1997). Characterization of urinary *Escherichia coli* O75 strains. J. Clin. Microbiol..

